# Relationship between Dyspnea Descriptors and Underlying Causes of the Symptom; a Cross-sectional Study

**Published:** 2017-03-19

**Authors:** Seyyed Mohammad Ali Sajadi, Alireza Majidi, Fahimeh Abdollahimajd, Fatemeh jalali

**Affiliations:** 1 Department of Internal Medicine, Ali-Ebne-Abitaleb Hospital, Rafsanjan University of Medical Sciences, Rafsanjan, Iran.; 2 Department of Emergency Medicine, Shohadaye Tajrish Hospital, Shahid Beheshti University of Medical Sciences, Tehran, Iran.; 3 Skin Research Center, Shahid Beheshti University of Medical Sciences, Tehran, Iran.

**Keywords:** Dyspnea, Asthma, Pulmonary Disease, Chronic Obstructive, Heart Failure, Symptom Assessment

## Abstract

**Introduction:**

History taking and physical examination help clinicians identify the patient’s problem and effectively treat it. This study aimed to evaluate the descriptors of dyspnea in patients presenting to emergency department (ED) with asthma, congestive heart failure (CHF), and chronic obstructive pulmonary disease (COPD).

**Method:**

This cross-sectional study was conducted on all patients presenting to ED with chief complaint of dyspnea, during 2 years. The patients were asked to describe their dyspnea by choosing three items from the valid and reliable questionnaire or articulating their sensation. The relationship between dyspnea descriptors and underlying cause of symptom was evaluated using SPSS version 16.

**Results:**

312 patients with the mean age of 60.96±17.01 years were evaluated (53.2% male). Most of the patients were > 65 years old (48.7%) and had basic level of education (76.9%). "My breath doesn’t go out all the way" with 83.1%, “My chest feels tight " with 45.8%, and "I feel that my airway is obstructed" with 40.7%, were the most frequent dyspnea descriptors in asthma patients. "My breathing requires work" with 46.3%, "I feel that I am suffocating" with 31.5%, and "My breath doesn’t go out all the way" with 29.6%, were the most frequent dyspnea descriptors in COPD patients. "My breathing is heavy" with 74.4%, "A hunger for more air” with 24.4%, and "I cannot get enough air" with 23.2%, were the most frequent dyspnea descriptors in CHF patients. Except for “My breath does not go in all the way”, there was significant correlation between studied dyspnea descriptors and underlying disease (p = 0.001 for all analyses).

**Conclusion:**

It seems that dyspnea descriptors along with other findings from history and physical examination could be helpful in differentiating the causes of the symptom in patients presenting to ED suffering from dyspnea.

## Introduction

History taking and physical examination help clinicians detect the patient’s problem and effectively treat it. Dyspnea is a subjective perception of difficulty breathing, commonly seen in patients with respiratory and cardiovascular diseases. Healthy subjects may also experience it in intense emotional states and during heavy exercise (-). 

Dyspnea is a multidimensional expression, which has been investigated extensively in clinical and psychological settings. Usually, it is identified as being unable to take a satisfying deep inspiration; moreover, it is characterized as difficulty breathing, which is described by air hunger and an uneasy awareness of breathing at rest or on exertion (4, 5). 

As with pain, different terms used to describe the sensation of dyspnea might indicate the underlying diseases (6). Previous studies have shown that descriptors are not only different among patients with different disorders but also in those with the same disease. For instance, to describe breathlessness, patients with asthma, prefer terms as "My chest feels tight "and "I cannot get enough air in", but patients with interstitial lung disease choose the phrase "My breathing is rapid" (-).

 The language used to describe dyspnea may be valuable in identifying the cause of dyspnea and choosing the best treatment modality (10). However, lots of variables such as linguistic and cultural differences can affect the results (6). The present study aimed to evaluate the relationship between different descriptors of dyspnea and underlying cause of the symptom in patients presenting to emergency department (ED) suffering from dyspnea. 

## Methods

***Study design and setting***

In this prospective cross-sectional study, all patients presenting to ED of Ali-Ebne-Abitaleb Hospital, Rafsanjan, Iran, with chief complaint of dyspnea, during 2 years, were evaluated regarding the descriptors of dyspnea. The study protocol was approved by Ethical Committee of Rafsanjan University of Medical Sciences. The patients were informed about all aspects of the research protocol and written informed consent was obtained from the patients. 

***Participants***

All patients aged 18 years and older presenting to ED of the mentioned hospital with chief complaint of dyspnea were enrolled using census sampling. Cases of communication disability (because of hearing problem, old age, illiteracy) and hemodynamic instability were excluded. Subjects were also excluded if an ultimate diagnosis other than congestive heart failure (CHF), chronic obstructive pulmonary disease (COPD), or asthma was reached. 

***Data gathering***

After initial assessment and emergency management, all patients were fully examined and eligible patients were included. A prepared questionnaire containing baseline characteristics and descriptors of dyspnea was used for data gathering. The questionnaire used here has been described in a previously published study (11). The questionnaire was translated into Persian language by the researchers and was evaluated in a pilot study, interviewing 20 patients and 5 emergency physicians, to confirm its validity and reliability (Cronbach's alpha for internal consistency = 0.86). To describe dyspnea, two trained medical students asked patients to choose three items from the list. An open ended question was also included allowing patients to describe their sensation if it was not represented in the questionnaire. The patients were categorized into three groups of CHF, COPD, and asthma based on final diagnosis, which was made based on imaging, spirometry, and other diagnostic tests needed. 

**Table 1:** Baseline characteristics of studied patients

**Variables**	**Number (%)**
**Age (year)**	
18 - 30	16 (5.1)
30 - 45	36 (11.5)
45 -65	108 (34.6)
≥ 65	152 (48.7)
**Sex**	
Male	166 (53.2)
Female	146 (46.8)
**Level of education**	
Basic	240 (76.9)
High school	64 (20.5)
University	8 (2.6)
**Final diagnosis**	
COPD	108 (34.6)
Asthma	118 (37.9)
CHF	86 (27.6)

CHF: congestive heart failure; COPD: chronic obstructive pulmonary disease.

**Table 2:** The relationship between different dyspnea descriptors and final diagnosis

Descriptors	Diagnosis n (%)			P
	Asthma	COPD	CHF	
**My breathing is shallow**	0 (0)	0 (0)	0 (0)	-
**I feel an urge to breathe More**	0 (0)	22 (20.4)	16 (18.6)	0.001
**My chest is constricted **	2 (1.7)	24 (22.2)	16 (18.6)	0.001
**My breathing requires effort **	2 (1.7)	22 (20.4)	6 (7)	0.001
**I feel a hunger for more air**	4 (3.4)	0 (0)	21 (24.4)	0.001
**I feel out of breath**	4 (3.4)	4 (3.7)	14 (16.3)	0.001
**I cannot get enough air**	0 (0)	0 (0)	20 (23.3)	0.001
**My breath does not go in all the way**	4 (3.4)	2 (1.9)	4 (4.7)	0.540
**My chest feels tight**	54 (45.8)	2 (1.9)	0 (0)	0.001
**My breathing requires work**	20 (16.9)	50 (46.3)	2 (2.3)	0.001
**I feel that I am suffocating/smothering**	40 (33.9)	34 (31.5)	12 (14)	0.004
**I feel that I cannot get a deep breath**	2 (1.7)	12 (11.1)	0 (0)	0.001
**I feel that I am breathing more**	0 (0)	2 (1.9)	8 (9.3)	0.001
**My breath does not go out all the way**	98 (83.1)	32 (29.6)	6 (7)	0.001
**My breathing is heavy**	4 (3.4)	2 (1.9)	64 (74.4)	0.001
**I feel that my airway is obstructed**	48 (40.7)	4 (3.7)	2 (2.3)	0.001

CHF: congestive heart failure; COPD: chronic obstructive pulmonary disease.

**Table 3:** The relationship between different dyspnea descriptors and level of education

Descriptors	Level of education n (%)			p
	Basic	High school	University	
**My breathing is shallow**	0 (0)	0 (0)	0 (0)	-
**I feel an urge to breathe more**	34 (14.2)	4 (6.2)	0 (0)	0.129
**My chest is constricted **	42 (17.5)	0 (0)	0 (0)	0.001
**My breathing requires effort **	24 (10.0)	6 (9.4)	0 (0.0)	0.639
**I feel a hunger for more air**	25 (10.4)	0 (0)	0 (0)	0.017
**I feel out of breath**	16 (6.7)	6 (9.4)	0 (0)	0.552
**I cannot get enough air**	16 (6.7)	4 (6.2)	0 (0)	0.749
**My breath does not go in all the way**	8 (3.3)	2 (3.1)	0 (0)	0.870
**My chest feels tight**	30 (12.5)	22 (34.4)	4 (50.0)	<0.001
**My breathing requires work**	50 (20.8)	20 (31.2)	2 (25.0)	0.212
**I feel that I am suffocating/smothering**	66 (27.5)	16 (25.0)	4 (50.0)	0.328
**I feel that I cannot get a deep breath**	12 (5.0)	2 (3.1)	0 (0)	0.670
**I feel that I am breathing more**	10 (4.2)	0 (0)	0 (0)	0.212
**My breath does not go out all the way**	86 (35.8)	42 (65.6)	8 (100.0)	<0.001
**My breathing is heavy**	66 (27.5)	4 (6.3)	0 (0)	<0.001
**I feel that my airway is obstructed**	24 (10)	24 (37.5)	6 (75.0)	<0.001

***Statistical analysis***

SPSS software version 16 was used for statistical analysis. Mean ± standard deviation or frequency and percentage were used for reporting the results. Chi square test was used to analyze relationships between different variables. Level of significance was 0.05 with a 95% confidence interval. 

## Results

312 patients with the mean age of 60.96±17.01 years (20 - 88) were evaluated (53.2% male). Table 1 shows the baseline characteristics of studied patients. Most of the patients were > 65 years old (48.7%) and had basic level of education (76.9%). The patients of all three groups (COPD, asthma, CHF) were similar regarding age, sex, and level of education (p > 0.05 for all analyses).

 "My breath doesn’t go out all the way" with 83.1%, “My chest feels tight " with 45.8%, and "I feel that my airway is obstructed" with 40.7%, were the most frequent dyspnea descriptors in asthma patients. 

"My breathing requires work" with 46.3%, "I feel that I am suffocating" with 31.5%, "My breath doesn’t go out all the way" with 29.6%, were the most frequent dyspnea descriptors in COPD patients. 

"My breathing is heavy" with 74.4%, "A hunger for more air” with 24.4%, and "I cannot get enough air" with 23.2%, were the most frequent dyspnea descriptors in CHF patients. 

Table 2 and 3 summarize the relation between different dyspnea descriptors with final diagnosis and level of education, respectively. Apart from “My breath does not go in all the way”, there was a significant correlation between studied dyspnea descriptors and final diagnosis (p = 0.001 for all analysis).

## Discussion

Based on the findings of the present study, patients prefer to use a variety of terminology to describe their sense of dyspnea. There was a significant relationship between the used terms and underlying cause of dyspnea and level of education. 

"My breath doesn’t go out all the way", "my breathing requires work" , and "my breathing is heavy", were the most frequent phrases used by asthma, COPD, and CHF patients, respectively, for description of their respiratory problem. 

The leading aim of understanding a patient’s dyspnea language is better diagnosis of underlying diseases and consequently increasing therapeutic efficacy. Different qualities of dyspnea sensation can point to prominent afferent mechanisms underlying clinical dyspnea making differential diagnosis easier and potentially advocate the best symptomatic therapy. 

More precise definition of symptoms in patients with shortness of breath has been looked into by researchers in the past. 

In a study, Williams et al. remarked that when the descriptors were not restricted to a single best word or phrase, individuals’ description of feeling breathless could differentiate people with and without a previous diagnosis of COPD (8). 

Several studies have shown that dyspnea perception among people is related to diverse etiologies including physiological, psychological, and racial causes and etc. (-). Some reports have stated that race, sex, educational level and socioeconomic class influence the perception of dyspnea (-). Barbaro et al. in a study designated that advanced age, airway inflammation, depression status, and severity of asthma affect perception of dyspnea (16).

The relationship between dyspnea descriptors and cause of symptom was strongly significant in the present study. In the study conducted by Mahler et al, the majority of patients with COPD applied work/effort descriptors such as “my breathing requires effort”; on the other hand, “I feel air hunger” had a lower prevalence in these patients (17). In addition, Chang et al. showed that the patients with asthma preferred “My chest feels tight” and mostly “Work/effort" descriptors were chosen by patients with COPD (18). 

In line with our findings, Rutgers et al. showed considerable differences in dyspnea perception between COPD and asthma (19). Caroci et al. noted that stable COPD and CHF patients prefer different terms to describe their breathing distress, however, they showed that they may use some similar terms (20). It seems that dyspnea descriptors along with other findings from history and physical examination could be helpful in differentiating the causes of the symptom in patients presenting to ED suffering from dyspnea. 

***Limitations***

The main limitation of our study was its relatively small sample size, also this study was conducted in a local region of Iran and its external validity may be limited. Therefore, the results cannot be generalized to the whole of Iranian population. Hence, further investigations are recommended with larger series to validate the findings. 

## Conclusion:

Based on the findings of the present study, patients prefer to use a variety of terminology to describe their sense of dyspnea based on underlying cause of symptom and level of education. It seems that dyspnea descriptors along with other findings from history and physical examination could be helpful in differentiating the causes of the symptom in patients presenting to ED suffering from dyspnea. 

## Author contribution:

Seyyed Mohammad Ali Sajadi was the lead author and contributed in study design, data gathering and manuscript preparation. Alireza Majidi was involved in concept development and study design and was involved in manuscript development. Fahimeh Abdollahimajd was involved in data gathering, data analysis, interpretation, and manuscript preparation and revision. Fatemeh jalali was involved in data gathering and interpretation.
